# Morphologic, Steroidogenic, and Transcriptomic Assessment of the Corpus Luteum in Holstein Cows after Spontaneous or Hormone-Induced Ovulation

**DOI:** 10.3390/ani13142283

**Published:** 2023-07-12

**Authors:** Patricio Ponce-Barajas, Marcos G. Colazo, Amir Behrouzi, Todd O. Ree, John P. Kastelic, Divakar J. Ambrose

**Affiliations:** 1 Department of Agricultural, Food and Nutritional Science, University of Alberta, Edmonton, AB T6G 1C9, Canada; patponceb@gmail.com (P.P.-B.); mgcolazo20@gmail.com (M.G.C.); behrouzi@ualberta.ca (A.B.); toddree77@gmail.com (T.O.R.); 2 Faculty of Veterinary Medicine, University of Calgary, Calgary, AB T2N 4Z6, Canada; jpkastel@ucalgary.ca

**Keywords:** corpus luteum, porcine LH, GnRH, estradiol benzoate, progesterone, induced ovulation, gene expression, luteal explants, histology

## Abstract

**Simple Summary:**

Poor reproductive efficiency in dairy cows is common due to low estrus detection, suboptimal fertility, and high embryonic losses. Synchronized ovulation protocols eliminate estrus detection and enable timed insemination. Refining such protocols for optimal outcomes is an ongoing endeavor. We previously reported that using a porcine luteinizing hormone in lieu of a gonadotropin-releasing hormone to synchronize ovulation significantly increased pregnancy per artificial insemination (AI) and altered intrafollicular protein milieu without affecting the blood concentrations of progesterone, a hormone essential for pregnancy maintenance in cattle. To elucidate underlying mechanisms, we compared the structure and function of corpora lutea that developed spontaneously or after hormone-induced ovulation in Holstein cows. Despite no differences in structure or gene expression, progesterone production by luteal explants was greater after ovulation induced with the porcine luteinizing hormone compared to other treatments. We inferred that augmented ovarian-level progesterone production likely increased uterine-level progesterone concentrations, which could promote embryo development and consequently increase pregnancy per AI.

**Abstract:**

There is evidence that replacing the gonadotropin-releasing hormone (GnRH) with porcine luteinizing hormone (pLH) to synchronize ovulation prior to artificial insemination (AI) increased pregnancy per AI in dairy cows without affecting blood progesterone (P_4_) concentrations. Whether morphologic, steroidogenic, and transcriptomic differences exist among corpora lutea (CL) formed after ovulation induced by GnRH and pLH is unclear. Our main objective, therefore, was to compare CL characteristics between GnRH- and pLH-induced CL. In 24 non-lactating Holstein cows, ovulations were spontaneous (Spont-Ov) or induced with 100 µg GnRH, 25 mg pLH, or 1 mg estradiol benzoate (EB), with CL excised 12 d after ovulation. In pLH- versus GnRH-treated cows, the duration of elevated LH (above baseline) was prolonged (10 versus 6 h, respectively, *p* < 0.01), but CL dimensions, pixel intensity of CL images, proportions of steroidogenic and non-steroidogenic luteal cells, and mean plasma LH did not significantly differ. Post-ovulation mean plasma P_4_ (ng/mL) did not differ among Spont-Ov (3.0) pLH (3.1) or GnRH (3.0) cows but were lower in EB cows (2.0). In vitro P_4_ concentration was greater in luteal explants of pLH-treated cows than in all other groups (combined means, 16.0 vs. 12.3 µg/mL, *p* < 0.02). Relative abundance of mRNA for oxytocin receptor (*OXTR*) was 2-fold higher (*p* < 0.01) in CL of pLH vs. GnRH cows and highest in Spont-Ov CL. In summary, pLH-treated cows had a longer LH peak, and greatest luteal tissue concentrations and in vitro production of P_4_. We inferred that increased P_4_ concentrations at the ovarian–uterine level in pLH-treated cows could have promoted embryo development and increased pregnancy per AI.

## 1. Introduction

Due to poor estrus detection efficiency in dairy cattle, various protocols for the synchronization of ovulation have been developed to enable timed artificial insemination (TAI) without estrus detection. One of the original approaches, the Ovsynch protocol [1], with two gonadotropin-releasing hormone (GnRH) treatments given 9 d apart and a prostaglandin F_2α_ (PGF) treatment given 7 d after the first GnRH, synchronizes ovarian follicular development, corpus luteum (CL) regression, and ovulation.

Although TAI pregnancies with the Ovsynch protocol are generally comparable to those after AI at a detected estrus [2,3,4], lower conception rates [5], higher embryonic losses [6] and a greater incidence of short estrous cycles implied compromised function of the CL after the second GnRH treatment [7,8]. These findings imply that cows subjected to the Ovsynch protocol develop a less functional CL consequent to the second GnRH treatment. In that regard, non-lactating Holstein cows induced to ovulate with GnRH had lower circulating progesterone concentrations than cows that ovulated spontaneously [9].

The duration of luteinizing hormone (LH) release after GnRH treatment is shorter (4 to 6 h) than a spontaneous preovulatory LH release (>10 h) [10,11]. Furthermore, cattle given porcine LH (pLH; [12,13,14]) or estradiol benzoate (EB; [15]) during proestrus also have a significantly longer duration of elevated LH concentrations than those given GnRH treatment. Therefore, using pLH or EB in lieu of GnRH to induce ovulation could improve CL function. In a study with 603 dairy cows, replacing the second GnRH with pLH in an Ovsynch protocol increased pregnancy per AI by 50% [16]. Although blood progesterone (P_4_) concentrations did not increase following pLH-induced ovulations in lactating [16] and non-lactating cows [13], the significant increase in pregnancy per AI in lactating dairy cows in the former study implied that cows benefitted from pLH. Regardless, how pregnancy per AI increased without increased peripheral P_4_ concentrations remains unclear. Bone morphogenetic protein 15, growth differentiation factor 9, and transforming growth factor-β1 were upregulated in the preovulatory follicle of pLH-treated cows [14], which increased the abundance of LH receptor, progesterone receptor, and cyclooxygenase-2 mRNA critical for ovulation and other reproductive functions. However, influences of pLH-induced ovulation on CL form and function are not known.

We hypothesized that there are morphologic, functional, and transcriptomic differences between CL induced by GnRH versus pLH in TAI protocols. Therefore, the primary objective was to determine whether CL characteristics, progesterone production (in vivo and in vitro), and the expression of genes regulating luteal function differed between GnRH- and pLH-induced CL in Holstein cows. A secondary objective was to compare the aforesaid end points in cows induced to ovulate following EB treatment, and those that underwent spontaneous ovulation (Spont-Ov).

## 2. Materials and Methods

This study was conducted at the Oscar Peterson Artificial Insemination Centre of Lakeland College (Vermilion, AB, Canada; 53°20′ N, 110°52′ W). Cows were group-housed and managed according to Canadian Council of Animal Care guidelines [17].

### 2.1. Cows and Experimental Design

**Figure 1:**
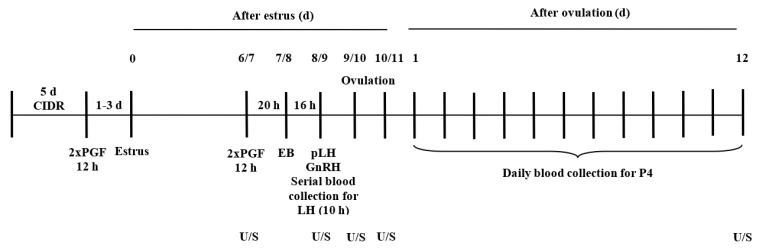
Schematic timeline of the study. Thirty Holstein cows were treated with a 1.9 g progesterone intravaginal device (CIDR) for 5 d and 500 µg cloprostenol (PGF) at CIDR removal. Estrus was detected thrice daily for 3 d. On days 6 or 7 (Estrus = Day 0), all cows were treated twice with PGF, and randomly allocated to1 of 4 treatment groups to receive: 100 μg GnRH, 25 mg pLH, 1 mg estradiol benzoate (EB), or no treatment (Spont-Ov). Estradiol benzoate was administered 20 h after the first PGF, whereas pLH or GnRH was given 16 h later. Plasma concentrations of LH and progesterone (P_4_) were determined in 28 and 24 cows, respectively. Transrectal ultrasonography (U/S) was used to determine CL dynamics, preovulatory follicle diameter, and ovulation.

Twenty-four cyclic, non-lactating Holstein cows were group-housed and given an intravaginal P_4_ device (CIDR, Zoetis Canada Inc., Kirkland, QC, Canada) for 5 d. Cows were then given two im injections of 500 μg cloprostenol (PGF; Estrumate, Merck Animal Health, Kirkland, QC, Canada), the first immediately after CIDR removal, followed by a second injection 12 h later. Cows were visually observed for estrus thrice daily for 30–60 min each time for 3 d, aided with Kamar^®^ heat detection patches (Kamar Inc., Steamboat Springs, CO, USA). On days 6 or 7 (Estrus = Day 0), all cows were again treated twice with PGF, 12 h apart, and allocated randomly and equally (n = 6/group) to one of four groups to receive intramuscular (im) injections of 100 μg GnRH (gonadorelin acetate, Fertiline, Vetoquinol N.-A. Inc., Lavaltrie, QC, Canada) 36 h after the first of two PGF [GnRH group]; 25 mg pLH (Lutropin-V, Vetoquinol N.-A. Inc.) 36 h after the first PGF [pLH group]; 1 mg estradiol benzoate (Sigma Chemical Co., St. Louis, MO, USA) in 1 mL canola oil 20 h after the first PGF [EB group]; or no additional treatment except the two PGF after CIDR removal, enabling spontaneous ovulations [Spont-Ov group] (Figure 1).

### 2.2. Transrectal Ultrasonography, CL Imaging and Blood Sampling

Transrectal ultrasonography (Aloka-SSD-500 scanner equipped with a 7.5 MHz linear-array transducer; Aloka Co., Tokyo, Japan) was performed at the first PGF treatment (6 or 7 d after estrus), and at 36 (at pLH and GnRH treatments), 72, and 96 h later to monitor ovarian structures and to confirm ovulation. Maximal diameter of the preovulatory follicle (POFD) was determined 36 h after the first PGF treatment (at pLH and GnRH treatments). Ovulation was confirmed by disappearance of a preovulatory follicle (diameter ≥ 10 mm) that had been detected at the previous examination [18]. Transrectal ultrasonography was also performed 12 d after ovulation to capture CL images for echotexture analysis and to determine CL diameter and area. The area of the CL was calculated using the equation: area = 0.5 height × 0.5 width × π, as described [19]. If a cavity was present, the luteal tissue area was calculated as area of entire CL minus area of cavity.

Image analysis of CL echotexture (12 d after ovulation) was conducted on video images using computer software (Pinnacle Studio, Version 8.45, Pinnacle Systems, Smart Sound^®^ Technology by Sonic Desktop, Real Producer SDK © 1995–2002 Real Networks, Inc., Mountain View, CA, USA). A single frame image from the video file, with the maximal CL diameter, was captured from the computer using an AVS Video Converter 5.6 (Online Media Technologies Ltd., London, UK). The entire area of the CL (excluding central cavity, if present) was analyzed (Scion Image for Windows, Frederick, MD, USA) for mean pixel intensity and heterogeneity [20]. Pixel intensity values are reported as a gray-scale value on a scale from 0 to 255, where 0 is black and 255 is white.

Sequential blood samples for LH determination were collected from GnRH- and pLH-treated cows, into 10 mL heparinized tubes (Vacutainer^®^, Beckton Dickson, Franklin Lakes, NJ, USA) via indwelling jugular catheters. Samples were collected 15 min prior to pLH or GnRH treatment, at the time of treatment, at 15 min intervals for 1 h after treatments, and then every 30 min for the next 9 h, for a total sampling period of 10 h and 15 min. Blood samples for P_4_ determination were collected once daily from 1 to 12 d after ovulation. Samples were stored in ice for up to 1 h, centrifuged (1500× *g*) for 20 min, plasma harvested and stored at −20 °C until hormone assays were performed.

### 2.3. Luteal Tissue Collection and Processing

Ovariectomy was performed by laparotomy on Day 12 postovulation using a left flank approach. Briefly, cows were sedated with an im injection of xylazine (0.05 mg/kg BW; Rompun, Provet, Lyssach, Switzerland) and an infiltrative local anesthesia with 2% lidocaine (Xylestesin; Bimeda-MTC Animal Health Inc., Cambridge, ON, Canada) was administered in the left paralumbar fossa. A flank incision was made to access the reproductive tract, and the ovary bearing the CL was removed using a chain écraseur. The peritoneum and external oblique muscle were closed with interrupted sutures and the skin was closed with simple sutures.

Immediately after removal, ovaries were placed in a 0.9% saline solution and taken to the laboratory. Within 10 min after being excised, each CL was blunt-dissected, weighed and split into equal halves by cutting perpendicular to the surface of the ovary using a surgical scalpel [2]. If a fluid-filled central cavity was evident upon cutting, the CL halves were reweighed to obtain net weight of the CL devoid of any fluid. A complete cross-sectional slice (2 to 4 mm thick) was cut from each half of the CL in a plane parallel to the cut surface of the ovary. One slice was further divided into smaller pieces and immediately placed in 2 mL cryogenic vials (CryowareTM, NALGENE^®^, Rochester, NY, USA), labeled, snap frozen and stored at −80 °C pending RNA extraction. The other slice was cut into smaller pieces (1 to 2 mm^3^), fixed in 10% buffered formalin, and stored for histological evaluations. The remaining CL tissues were processed for in vitro culture.

### 2.4. In Vitro Culture for Progesterone

Approximately 250 mg of CL tissue (3–4 mm cubes) was weighed, washed three times in Dulbecco’s Modified Eagle’s Medium (DMEM; Gibco^®^ DMEM, Life Technologies Inc., Burlington, ON, Canada), placed in 6 mL of pre-warmed DMEM (in duplicate) and cultured for 2 h at 38.5 °C in 5% CO_2_ atmosphere with 0, 20 or 40 ng/mL of bovine LH (Sioux Biochemical Inc., Sioux Center, IA, USA) as described [2]. An additional control group was included (also in duplicate) wherein 6 mL of cold (4 °C) absolute ethanol was added to the culture dish containing the CL tissue and DMEM to stop progesterone synthesis in vitro, followed by no incubation. At the end of the 2 h incubation period, 6 mL of cold absolute ethanol was added to each culture dish to stop further progesterone synthesis, and samples including the DMEM, CL tissue and ethanol were stored at −20 °C until progesterone concentrations were determined. The no-incubation control samples were frozen immediately after cold ethanol was added.

### 2.5. Histological Evaluation

Formalin-fixed CL tissues were dehydrated in a series of alcohol baths and embedded into individual paraffin blocks. Then, 10 μm sections from each block were cut, mounted on clean glass slides, and stained with haematoxylin and eosin [21]. Two sections from each CL were then randomly selected, photographed at 400×, and captured images printed in colour. Cells from within two 10 × 10 cm grids of each photograph were counted by two individuals. On average 276 ± 69 (SD) cells were present in each grid; thus, ≈1104 cells were counted from each CL, and relative proportions of steroidogenic luteal cells (large and small luteal cells) and nonsteroidogenic cells (endothelial cells and fibroblasts) were calculated as a percent of total cells and compared for statistical differences.

### 2.6. Hormone Assays

#### 2.6.1. P_4_ Assays

Plasma P_4_ concentrations were determined using a modified, solid-phase ^125^I-radioimmunoassay kit (Coat-A-Coat^®^ Progesterone; Diagnostic Products Corporation, Los Angeles, CA, USA). The sensitivity was 0.1 ng/mL, with intra- and inter-assay coefficients of variation of 6.2 and 9.8%, respectively.

After homogenizing the CL tissue, P_4_ was extracted as described (Schmitt et al., 1996 [2]). Procedural losses were estimated by adding tritiated [^3^H] progesterone before homogenization. Total progesterone content of CL tissue and secreted progesterone in culture medium were determined by Coat-a-Count^®^ radioimmunoassay kit, after a 1:200 dilution. Concentrations were corrected for the dilution effect, adjusted for the exact weight of CL tissue, and reported in μg/mL.

#### 2.6.2. LH Assay

Plasma LH concentrations were measured by radioimmunoassay, using an anti-bovine LH monoclonal antibody (518B7; Quidel Corporation, San Diego, CA, USA; provided by Dr. Janet Roser, Department of Animal Science, University of California-Davis). This antibody cross-reacted equally with bovine and porcine LH [22]. The LH peak was defined as the highest LH concentration, and the mean LH concentration (ng/mL) was the average of all samples from GnRH or pLH treatment up to 10 h post-treatment. In GnRH-treated cows, the duration (h) of LH surge was the interval from GnRH treatment to the return of LH to pre-treatment basal concentrations (mean of the two samples taken before GnRH administration).

### 2.7. Transcriptomics

#### 2.7.1. RNA Extraction

Luteal tissue obtained from 19 cows (GnRH, n = 4; pLH, n = 6; EB, n = 5; Spont-Ov, n = 4) were used. The CL pieces were pulverized (using pestle and mortar) under liquid nitrogen, and 100 mg of tissue was weighed and transferred into 1.5 mL RNAse- and DNAse-free microfuge tubes (Life Technologies, Burlington, ON, Canada). Total RNA was extracted using TRIzol^®^ Plus system (12183555-Invitrogen, Carlsbad, CA, USA), subjected to RNase-Free DNase treatment (QIAGEN Inc., Mississauga, ON, Canada), and purified with an RNA spin cartridge system (QIAGEN Inc.) according to the manufacturers’ instructions. The RNA was treated with RNase inhibitor (20 U/L—Applied Biosystems, Streetsville, ON, Canada) and quantified using a NanoDrop microvolume spectrophotometer (ND1000—NanoDrop Technologies, Wilmington, DE, USA).

#### 2.7.2. RT-qPCR

To synthesize cDNA, 1 µg of RNA was reverse-transcribed using TaqMan^®^ reverse transcription reagents (Applied Biosystems, Streetsville, ON, Canada), random hexamers (50 μM), and oligo d(T)16 primers.

Primer sequences and Taqman-MGB probes for each gene were designed with Express^®^ software v3.0 (Applied Biosystems Inc., Foster City, CA, USA) based on species-specific sequences in GENBANK (Table 1). Real-Time PCR was performed in triplicates in 96-well plates using the Taqman^®^ Universal PCR Master Mix (Applied Biosystems Inc.) and the ABI 7900HT thermocycler (Applied Biosystems., Inc.). The real-time PCR program parameters were as follows: 95 °C for 20 min, then 40 cycles of 95 °C for 10 min and 60 °C for 60 s. The comparative cycle threshold (CT) method was used to calculate accurate and reproducible data for relative gene expressions [23]. Briefly, the relative gene expressions of targeted genes were normalized against housekeeping genes (H2A histone family member Z, *H2AZ1*; glyceraldehyde-3-phosphate dehydrogenase, GAPDH; and succinate dehydrogenase complex, subunit A flavoprotein, SDHA) per individual. Consequently, relative changes in gene expression were analyzed using the 2^−ΔΔCT^ method [24].

### 2.8. Statistical Analyses

The Statistical Analysis System (SAS Version 9.1 for Windows; SAS Institute, Cary, NC, USA) software was used for data analyses. Data were tested for normal distribution (PROC univariate) prior to employing a test of significance.

Plasma LH and progesterone concentrations were analyzed by repeated measures using the MIXED procedure of SAS (9.1; SAS Institute Inc., Cary, NC, USA) with an autoregressive covariance structure and the time of blood sample collection as the repeated effect. The statistical model used was *Yijk* = *μ* + *Ti* + *Pj* + *C* (*T*)*ik* + *εijk*, where *Yij* is the individual observation, *μ* is the overall mean, T*i* is the effect of treatment (*i* = 1, 2), and *Pj* is the effect of time period (*j* = 1, 2, 3 … and 10). The term *C* (*T*)*ik* was included as a random effect (*k* = 1, 2, 3 and 4; treated as a random effect), and ε*ijk* is the residual error term. Cow effect was included in the model as a random effect.

Ovulation responses to treatments were compared among groups using a Fisher’s Exact test. Preovulatory follicle diameter, CL diameter, and area, pixel intensity and P_4_ concentration 12 d after ovulation were analyzed by one-way ANOVA, using Bartlett’s test to confirm equality of variance. Treatment effects were determined for comparisons assessed regarding the individual fold-change for each gene of interest. Data were analyzed by ANOVA using a MIXED procedure, and *p* < 0.05 was considered significant. The statistical model was *Y_ij_* = *μ* + *T_i_* + *C* (*T*)*_ij_* + *ε_ij_*, where *Y_ij_* is the individual observation, *μ* is the overall mean, *T_i_* is the effect of treatment (*i* = 1, 2). The term *C* (*T*)*_i__j_* was included as a random effect (*j* = 1, 2, 3 and 4; treated as a random effect), and ε*_ij_* is the residual error term.

Progesterone concentration data from the in vitro culture study were analyzed by repeated measures using the MIXED procedure of SAS with an autoregressive covariance structure. Pre-incubation P_4_ concentrations were used as covariate factors.

## 3. Results

Of the 24 cows, 1 cow assigned to the GnRH treatment group did not respond to PGF treatments and hence was excluded from the study. Three cows (EB = 1, Spont-Ov = 2) failed to ovulate and 1 GnRH-treated cow had a short estrous cycle by regressing her CL prior to 12 d after ovulation. Therefore, data from only 19 cows are presented except for LH concentrations, which are reported for 11 cows (GnRH, n = 5 and pLH, n = 6).

### 3.1. Ovulation and CL Morphology

All GnRH- and pLH-treated cows ovulated, whereas only 5 of 6 EB-treated cows and 4 of 6 Spont-Ov cows ovulated. The ovulatory responses as determined by ultrasonography are shown in Table 2. The POFD was larger (*p* < 0.05) in Spont-Ov cows than in cows from all other treatment groups (Table 2). The mean diameter of the CL 12 d after ovulation was largest in Spont-Ov cows, intermediate in cows treated with pLH or EB, and smallest in cows given GnRH. However, neither the CL area nor mean pixel intensity of the captured CL images differed among treatment groups (all data shown in Table 2).

The mean CL weight was lower (*p* < 0.05) in cows induced to ovulate with GnRH (4.5 g) than in Spont-Ov cows (6.1 g), but it did not differ from other treatment groups (Table 2).

### 3.2. Plasma P_4_ and LH Concentrations

**Figure 2:**
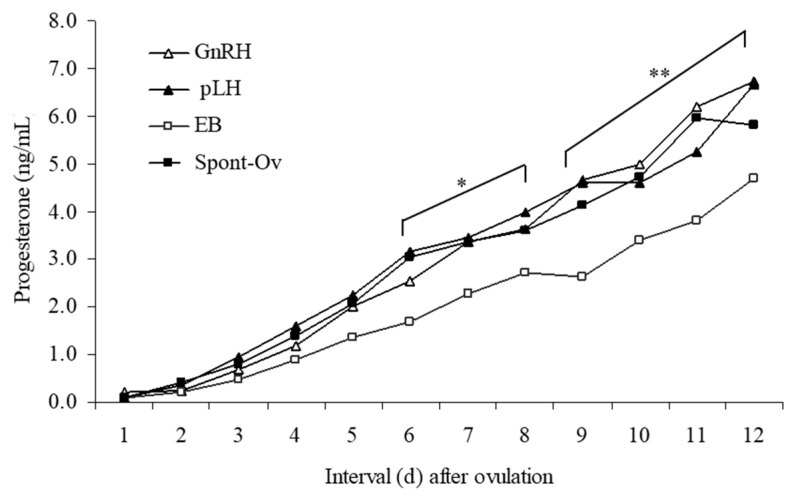
Mean plasma progesterone (P_4_) concentrations in non-lactating Holstein cows given 100 µg GnRH (n = 4), 25 mg pLH (n = 6), 1 mg estradiol benzoate (EB; n = 5), or no treatment (Spont-Ov, n = 4) following the administration of PGF. One cow that had a short estrous cycle after treatment with 100 µg GnRH was excluded. Plasma P_4_ concentrations were affected by treatment and time (*p* < 0.01). Mean plasma P_4_ in cows treated with 100 µg GnRH or 25 mg pLH did not differ from that in Spont-Ov cows. However, cows treated with 1 mg EB had lesser plasma P_4_ concentrations. Treatment x time interaction tended (*p* = 0.09) to affect P_4_ concentrations. The pooled SEM were 0.65, 0.60, 0.43, and 0.58 ng/mL for GnRH, pLH, EB and Spont-Ov, respectively. * GnRH, pLH and Spont-Ov > EB, *p* = 0.08. ** GnRH, pLH and Spont-Ov > EB, *p* < 0.05.

Plasma P_4_ concentrations after ovulation differed (*p* < 0.05) among treatment groups (Figure 2). Mean P_4_ concentrations were lowest in cows given EB (*p* < 0.05) but did not differ among the other three groups.

**Figure 3:**
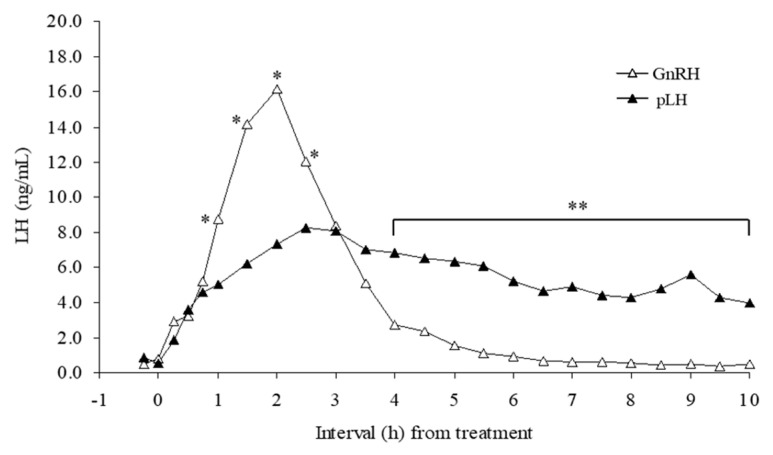
Mean plasma LH concentrations from cows treated with 100 μg of the gonadotropin-releasing hormone (GnRH; n = 5) or 25 mg of the porcine luteinizing hormone (pLH; n = 6). Mean LH concentrations did not differ by treatment (*p* > 0.05) but there was a time effect, and a treatment x time interaction (*p* < 0.01). The pooled SEM were 0.95 and 0.41 ng/mL for GnRH and pLH treatments, respectively. * GnRH > pLH, *p* < 0.01; ** pLH > GnRH, *p* < 0.01.

Mean plasma LH concentrations (ng/mL) did not differ between pLH- (5.0 ± 0.5) and GnRH-treated (3.8 ± 0.5) cows; however, there were effects of time and a treatment-by-time interaction (*p* < 0.01; Figure 3). In GnRH-treated cows, LH peaked by 2 h (16.2 ng/mL), returning to basal concentrations (0.7 ng/mL) by 6 h. In pLH-treated cows, however, LH increased from 0.5 ± 0.1 ng/mL to 8.25 ± 1.7 ng/mL (*p* < 0.05) by 2.5 h after pLH treatment and did not return to basal concentrations during the 10 h blood sampling period.

### 3.3. In Vitro Culture and P_4_ Production

#### 3.3.1. In Vitro P_4_ Production

**Figure 4:**
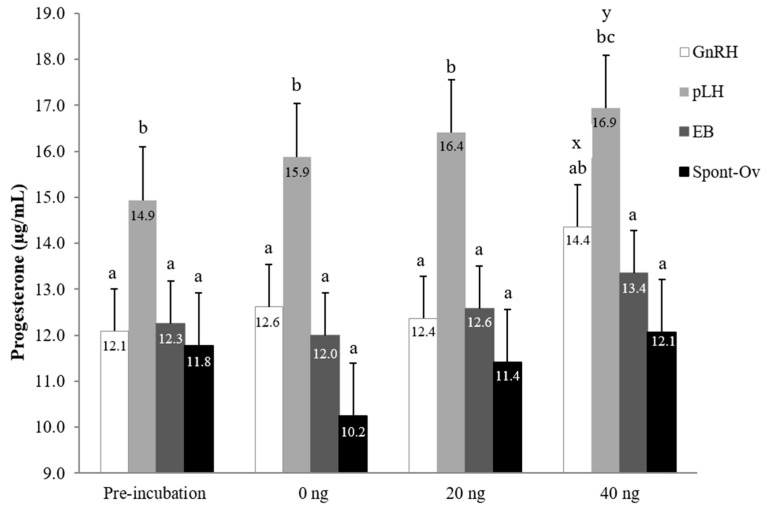
In vitro P_4_ concentrations (µg/mL) from CL tissue and cultured media. Progesterone from CL tissue (pre-incubation) and cultured media (secreted P_4_ in response to the addition of bovine LH at 0, 20 or 40 ng levels to the culture medium) in cows given GnRH (100 µg), pLH (25 mg), estradiol benzoate (EB; 1 mg), or no treatment (Spont-Ov) following PGF. ^a–c^ Bars without a common superscript within each cluster differ (*p* < 0.05). ^x,y^ *p* = 0.07.

Progesterone from CL tissue and culture media were measurable in all treatment groups. Both in vivo treatment and the addition of bovine LH to culture medium increased the P_4_ concentration in vitro, but there was no interaction between treatment and bovine LH dose. The combined average P_4_ concentration (mean ± SEM; µg/mL), in vitro, in pLH treatment (16.03 ± 0.98) was greater (*p* < 0.02) than that of GnRH (12.88 ± 0.71), EB (12.58 ± 0.69) and Spont-Ov (11.38 ± 0.97) treatments (Figure 4). Mean in vitro P_4_ concentrations did not differ (*p* > 0.32) among non-incubated control (12.76 ± 0.36), 0 ng LH (12.68 ± 0.36) and 20 ng LH (13.19 ± 0.36). However, the P_4_ concentration of the 40 ng LH dose (14.18 µg/mL) was greater (*p* < 0.01) than that of the non-incubated control and 0 ng LH treatment incubated for 2 h, and tended (*p* < 0.07) to be greater than that of the 20 ng dose. Within each of the four groups (i.e., non-incubated control, 0, 20, and 40 ng LH-supplemented groups incubated for 2 h), P_4_ concentrations were consistently greater (*p* < 0.05) in the luteal tissue obtained from pLH-treated cows.

#### 3.3.2. Histological Evaluation

Relative proportions of steroidogenic luteal cells and non-steroidogenic luteal cells in bovine CL after induced or spontaneous ovulation did not differ among treatments (Table 3).

### 3.4. mRNA Expression in CL Tissue

Molecular assessments of CL for expressions of mRNA levels of genes associated with steroidogenesis and other CL functions measured by RT-PCR are presented (Table 4). Whereas *STAR, CYP11A1* and *SREBF1* are involved in luteal steroidogenesis, their relative mRNA abundance did not differ among treatments. The relative abundance of luteotropic genes PGE receptor (*PTGER2*), glucocorticoid receptor (*NR3C1*), Pit-Oct-Unc class 5 homeobox 1 (*POU5F1*), and a luteolytic gene, prostaglandin F2 α receptor (*PTGFR*), all associated with CL viability and survival, did not differ among treatments. Relative abundance of mRNA for oxytocin receptor gene *OXTR* was approximately 2-fold higher in the luteal tissue of pLH-treated cows vs. GnRH-treated cows (*p* < 0.01); however, Spont-Ov cows had the highest expression of *OXTR* among all four treatments.

## 4. Discussion

Although newer TAI protocols that can yield greater pregnancy per AI than after AI at detected estrus are now available [25], they are more complex, of longer duration, and prone for errors during implementation because of increased interventions. The original Ovsynch protocol is easy to implement and relatively less expensive. Therefore, attaining greater pregnancy per AI by using pLH in lieu of GnRH to synchronize ovulation is a desirable option. In that regard, the current study was an attempt to understand the basis of a reported increase in pregnancy per AI in lactating dairy cows [16], when ovulation was synchronized by pLH vs. GnRH in a TAI protocol (42 vs. 28%, respectively, *p* < 0.05). Despite the significant increase in pregnancy per AI, blood progesterone concentrations were not different, implying that the increased pregnancy per AI in pLH-treated cows was likely due to a localized effect at the utero-ovarian level. Therefore, the present investigation focused on potential differences in CL competence at various levels, including morphologic (CL size, weight, pixel intensity, and histology), steroidogenic (systemic and luteal progesterone concentrations) and transcriptomic (mRNA expression). As costs associated with the use of lactating cows are prohibitive, we used non-lactating dairy cows as our model and obtained mature CL 12 d after confirmed ovulation. Although the primary emphasis of the present study was to compare CL morphology and function after the synchronization of ovulation using pLH versus GnRH, we also made comparisons with CL from spontaneous and EB-induced ovulations.

The POFD did not differ between GnRH and pLH-treated cows, but the mean POFD of GnRH-, pLH- and EB-treated cows was significantly smaller than that of Spont-Ov cows. Similarly, cows given 250 μg of a GnRH analogue had a smaller POFD compared to spontaneously ovulating cows when measured before ovulation [9]. Changes in luteal echotexture, as determined by the measurement of pixel values (brightness of picture elements) of ultrasound images of corpora lutea, were associated with luteal function during the estrous cycle [26]. In the current study, the mean CL diameter, area, pixel intensity and CL weight (determined after dissection) were also not different between cows that were induced to ovulate with either GnRH or pLH, but the CL diameter and weight were significantly lower in GnRH-treated cows than in Spont-Ov cows.

Postovulatory P_4_ concentrations did not differ between GnRH- and pLH-treated cows, corroborating previous findings [13,16]. Although it has been reported that EB-induced ovulations are followed by normal CL [27], in the present study, P_4_ concentrations were significantly lower in EB-treated cows than all other treatment groups. Neither POFD nor CL morphology differed among EB-treated cows and those given GnRH or pLH. The POFD, however, was larger in the Spont-Ov group than in all other treatment groups, and the CL weight was significantly lower in the GnRH group compared to the Spont-Ov group. Although Spont-Ov cows had larger POFDs, they did not have significantly larger CL (except vs. GnRH treatment) or higher P_4_ concentrations. In another study, although the diameter of CL on Day 10 did not differ in cows induced to ovulate with either EB or GnRH compared to the control group (Spont-Ov), the maximum plasma P_4_ concentration on Day 9 and P_4_ rise were lower in both GnRH and EB treatments compared to the Spont-Ov group [9].

Mean plasma LH concentrations did not differ between pLH- and GnRH-treated cows. However, the LH peak was significantly higher in the GnRH-treated group, and plasma LH concentrations remained above baseline throughout the 10 h blood sampling period in pLH-treated cows. Although comparable LH concentrations of EB and Spont-Ov groups were not available, it has been reported that a preovulatory LH surge induced by exogenous estradiol was similar to a spontaneous preovulatory LH surge [28], and that EB-induced ovulation in TAI protocols yielded acceptable pregnancy per AI [27]. Furthermore, heifers given saline (control) in CIDR-based Ovsynch protocols had a much more variable LH pattern, with spontaneous LH surges occurring from 8 to 30 h after treatment [12].

Our earlier finding [16] that a substantial improvement in pregnancy per AI occurred without a corresponding increase in peripheral blood progesterone concentrations raised the possibility that pLH-induced CL produces more P_4_ locally. Uterine tissue P_4_ concentrations are driven by the P_4_ content of CL [29], and P_4_ distributions in the bovine uterus, broad ligament and uterine arteries are preferentially increased on the side ipsilateral to the ovary bearing the CL [30]. A P_4_ gradient within these tissues has been reported [30], with significantly greater concentrations of P_4_ in tissues closest to the ovary bearing the CL (e.g., mesosalpinx, anterior uterine horn, and branches of the uterine artery supplying that area) than in more distal tissues or the main uterine artery. Notably, no such P_4_ gradient was evident in tissues on the side contralateral to the ovary bearing the CL in that study [30]. Although our study was not designed to assess P_4_ gradients, we addressed the question of whether CL induced after pLH compared to GnRH treatment contained higher concentrations of P_4_ within the luteal tissue, and whether explants of pLH-induced CL secreted higher quantities of P_4_ in vitro. Luteal P_4_ concentrations were significantly higher in pLH-treated cows even at time zero (pre-incubation), clearly indicating that P_4_ secretion from luteal tissue was greater in pLH-treated cows. Control (pre-incubation) samples were representative of P_4_ within the CL tissue soon after surgical excision, whereas P_4_ concentrations after 2 h in culture were representative of total P_4_ (i.e., P_4_ in tissue at harvest plus P_4_ secreted during culture). Although P_4_ concentrations did not differ between pre-incubation and post-incubation (0 ng) samples, increased P_4_ after the addition of LH, particularly after 40 ng/mL, indicated that the in vitro production of P_4_ by luteal tissue was dependent on exogenous LH during culture. A dose-dependent increase in LH production in vitro was most evident in CL tissue from pLH-treated cows.

Despite no significant differences in luteal cell numbers among treatments at histology, perhaps small luteal cells, which have LH receptors, were larger, as reported previously by Schmitt et al. [2], contributing to increased P_4_ production.

We also assessed CL function at the molecular level, indicated by expression levels of genes associated with CL steroidogenesis and viability. We selected a set of genes that regulate steroidogenesis (*STAR*, *CYP11A*, *SREBF1*) and luteal viability and life span (angiogenesis, luteotropic and luteolytic activities, and transcription).

A balanced expression of steroidogenic, luteotropic, luteolytic and apoptotic genes determines CL function at the molecular level [31,32,33,34]. *STAR*, *CYP11A*, and *SREBF1* represent >98% of protein-encoding genes associated with P_4_ biosynthesis [35]. Vascular endothelial growth factors (*VEGFA*) are regulators of luteal angiogenesis and support formation and sustenance of a viable CL [36]. Luteal maintenance in cattle is controlled by luteotropic (*PGE* receptor, *POU5F1*) and luteolytic genes, mainly *OXTR*, *COX-2*, *PTGFR*, *PPARG* and *FAS* [37]. Oxytocin receptor (*OXTR*), an important regulator of the luteolytic cascade, triggers the end of the luteal phase [38,39]. The significance of the increased *OXTR* mRNA expression in pLH and Spont-Ov cows compared to GnRH and EB groups in the present study is not known. In a previous study, there was no significant relationship between the expression of genes of importance to CL function, including *OXTR*, and mid luteal P_4_ concentrations [40]; therefore, the relevance of differential expressions of *OXTR* in our study remains undetermined. The previous study [40] reported that plasma P_4_ concentrations in the mid-luteal phase were dependent on luteal size, but independent of blood flow and gene expression per luteal tissue unit.

It is well documented that LH triggers a cascade of signaling networks in the preovulatory follicle, culminating in the ovulation of a mature oocyte and improving its competence [41]. The LH surge can both directly and indirectly initiate the activation of the G-protein-coupled receptors (*GPCR*) and epidermal growth factor (*EGF*) network [42]. Stimulation of LH induces the rapid and transient expression of EGF family members and LH receptors in mural granulosa cells and cumulus cells, inducing oocyte maturation and improving oocyte competence. In a report from our research group [14], the relative abundance of *BMP-15*, *GDF-9* and *TGF-β1* in the follicular fluid of preovulatory follicles was significantly higher in pLH- versus GnRH-treated cows.

## 5. Conclusions

The current report presents new evidence that pLH-treated cows produce more P_4_ at the luteal level. Plasma P_4_ concentrations did not differ between pLH- and GnRH-treated cows, yet P_4_ concentrations in the luteal tissue of pLH-treated cows were greater, thus implying that increased P_4_ production at the luteal level may be preferentially utilized by uterine tissue. Greater P_4_ concentrations at the uterine level could promote embryo growth and elongation, leading to larger embryos and more robust signaling at the time of pregnancy recognition, resulting in increased pregnancy per AI. Collectively, our previous report [14], that pLH-treated cows secreted more intrafollicular *BMP-15*, *GDF-9* and *TGF-β1*, which could improve oocyte competence, and the present finding, that the CL tissue of pLH-treated cows secreted more P_4_, indicated that the greater pregnancy per AI in pLH-treated cows than in GnRH-treated cows [16] may have occurred through more than one mechanism.

## Figures and Tables

**Table 1:** Primers designed for genes of interest in the CL tissue of Holstein cows 12 d after spontaneous or induced ovulation.

Gene Name	Forward Sequence (5′-3′)	Reverse Sequence (5′-3′)	Accession No.	Amplicon (bp)
*STAR*	5′-AGAAGGGTGTCATCAGAGCG-3′	5′-ATCCCTTGAGGTCAATGCTG-3′	NM_174189	114
*CYP11A1*	5′-ACCTGATTCCTGCCAAGACAC-3′	5′-AGAATGTGGATGAGGAAGAGG-3′	NM_176644	220
*SREBF1*	5′-TGCAGACCCTGGTGAGTGG-3′	5′-AGATTTATTCAACTTGGCCTCGG-3′	NM_001113302	236
*VEGFA*	5′-ACATCACCATGCAGATTATGCG-3′	5′-ACAGGGATTTTCTTGCCTTGC-3′	NM_174216	128
*OXTR*	5′-AGGAAGCCTCACCTTTCATCATC-3′	5′-AGCGCTGCACAAGTTCTTGG-3′	NM_174134.2	114
*PTGS2*	5′-AGGGCTGGCAGGGTCG-3′	5′-AGCCATTTCCTTCTCTCCTGTAAG-3′	NM_174445	177
*PTGFR*	5′-TTCATTTGTTTGCAATGCCATC-3′	5′-TGGCCATTGTCACCAGAAAAG-3′	NM_181025	167
*PTGER2*	5′-TTCAGTGTCATCGTCAACCTCATC-3′	5′-ATATATGCAAAAATCGTGAAAGGCA-3′	NM_174588	194
*NR3C1*	5′-ACCTTACTGCTCCTCTCTTC-3′	5′-TTCAACCACTTCATGCATAG-3′	NM_001206634	189
*FAS*	5′-TGCACCACGTGTGAACATG-3′	5′-TTGCCTCCCTTCATCATTTG-3′	NM_174662	211
*POU5F1*	5′-TGGAGGAAGCTGACAACAACG-3′	5′-AAAACCACACTCGGACCACG-3′	NM_174580	213
*PPARG*	5′-TGCAAGGACCTCACAAGAAATTAC-3′	5′-TGCACTTTGGTACTCTTGGAGC-3′	NM_181024	250
*H2AZ1*	5′-ACCGCAGAGGTACTTGAATTGG-3′	5′-TGGAATGACACCACCACCAG-3′	NM_174809	150
*GAPDH*	5′-ACAACACCCTCAAGATTGTCAGC-3′	5′-TGGCGTGGACAGTGGTCATA-3′	NM_001034034	120
*SDHA*	5′-AGGACTTCAAGGAGAGGGTTGAC-3′	5′-TCCAGGGTGACCTTCCCAG-3′	NM_174178	136

**Table 2:** Ovarian responses in non-lactating Holstein cows given GnRH (100 µg), pLH (25 mg), estradiol benzoate (EB; 1 mg), or no treatment (Spont-Ov) after the administration of PGF.

		GnRH	pLH	EB	Spont-Ov
No. of cows		5	6	6	6
Ovulatory response	No. of cows	5	6	5	4
	%	100	100	83.3	66.6
Preovulatory follicle	Diameter (mm)	14.3 ± 1.0 ^b^	15.2 ± 0.8 ^b^	15.6 ± 0.8 ^b^	18.4 ± 0.9 ^a^
Corpus luteum ^1^	Diameter (mm)	20.4 ± 1.2 ^b^	22.7 ± 0.9 ^ab^	22.5 ± 1.0 ^ab^	25.1 ± 1.2 ^a^
	Area ^2^ (mm^2^)	356.9 ± 44.9	451.0 ± 36.7	392.8 ± 40.2	364.0 ± 44.9
	Pixel intensity				
	Mean	148.7 ± 4.0	156.0 ± 3.3	155.9 ± 3.6	158.6 ± 4.0
	Weight (g)	4.5 ± 0.5 ^b^	5.4 ± 0.4 ^ab^	5.2 ± 0.4 ^ab^	6.1 ± 0.5 ^a^
Postovulatory progesterone (ng/mL) ^1^	Mean, 1 to 12 d	3.0 ± 0.2 ^a^	3.1 ± 0.2 ^a^	2.0 ± 0.2 ^b^	3.0 ± 0.2 ^a^
	Mean, at 12 d	6.7 ± 0.6 ^a^	6.7 ± 0.5 ^a^	4.7 ± 0.5 ^b^	5.6 ± 0.6 ^ab^

^ab^ Within a row, means without a common superscript differed (*p* < 0.05). ^1^ One cow that had a short estrous cycle after treatment with 100 µg GnRH was excluded. ^2^ CL area measurements were adjusted by subtracting the area of the central cavity.

**Table 3:** Relative proportion of steroidogenic luteal cells and non-steroidogenic luteal cells in bovine CL developed following induced or spontaneous ovulation (least squares mean ± SEM).

Treatment	SLC ^1^	NSLC ^2^
GnRH	45.20 ± 0.59	54.80 ± 0.59
pLH	43.53 ± 0.59	56.47 ± 0.59
EB	42.24 ± 0.49	57.76 ± 0.49
Spont-Ov	47.73 ± 0.37	52.27 ± 0.38

^1^ SLC = steroidogenic luteal cells (large and small luteal cells combined, expressed as % of total cells per unit area, based on ≈1104 cells counted per CL). ^2^ NSLC = non-steroidogenic luteal cells (endothelial cells and fibroblasts combined, expressed as % of total cells per unit area, based on ≈1104 cells counted per CL).

**Table 4:** Quantitative real-time PCR analysis of genes associated with luteal function (CL viability, survival and steroidogenesis) abundant in the CL of non-lactating Holstein cows induced to ovulate after GnRH (100 µg), pLH (25 mg), estradiol benzoate (EB; 1 mg) treatment, or no treatment (Spont-Ov).

	Treatment				*p* Value
	GnRH (n = 4)	pLH (n = 6)	EB (n = 5)	Spont-Ov (n = 4)	
*STAR*	1.1 ± 0.1	0.9 ± 0.1	1.1 ± 0.1	0.7 ± 0.1	0.27
*CYP11A1*	0.8 ± 0.2	0.9 ± 0.1	1.0 ± 0.2	0.6 ± 0.2	0.70
*SREBF1*	0.9 ± 0.2	0.7 ± 0.2	0.4 ± 0.2	0.4 ± 0.2	0.67
*VEGFA*	0.5 ± 0.1	0.4 ± 0.1	0.4 ± 0.1	0.3 ± 0.1	0.71
*OXTR*	0.3 ± 0.1 ^b^	0.6 ± 0.1 ^a^	0.2 ± 0.1 ^b^	0.9 ± 0.1 ^a^	0.01
*PTGS2*	0.9 ± 0.2	0.5 ± 0.2	0.5 ± 0.2	0.5 ± 0.2	0.67
*PTGFR*	1.0 ± 0.2	1.1 ± 0.1	1.0 ± 0.1	0.8 ± 0.1	0.59
*PTGER2*	1.0 ± 0.2	0.7 ± 0.1	0.94 ± 0.1	0.5 ± 0.2	0.38
*NR3C1*	1.2 ± 0.2	1.1 ± 0.1	1.3 ± 0.2	1.2 ± 0.2	0.96
*FAS*	0.9 ± 0.2	0.8 ± 0.1	1.0 ± 0.2	0.8 ± 0.2	0.96
*POU5F1*	0.5 ± 0.2	0.8 ± 0.1	0.5 ± 0.2	0.5 ± 0.2	0.60
*PPARG*	0.007 ± 0.1	0.08 ± 0.1	0.4 ± 0.1	0.06 ± 0.1	0.13

^a,b^ *p* < 0.01.

## Data Availability

Data are contained within the article.
